# Clinical Observations on the Diseases of New-Born Infants

**Published:** 1839-01

**Authors:** 


### ART. III.

1. *Clinique des Maladies des Enfans Nouveau-nés.* Par F. L. I. VALLEIX, M.D., Membre titulaire de la Société Médicale d'Observation de Paris, &c.—*Paris*, 1838.

*Clinical Observations on the Diseases of New-born Infants.* By F. L. I. VALLEIX, M.D., &c.—*Paris*, 1838.

2. *De Tumore Cranii recens Natorum sanguineo Symbolæ. Quibus, viro experientissimo* ELIÆ HERSCHEL, M.D., &c. gratulatur JOANNES AUGUSTUS BURCHARD, M.D., &c.—*Vratislaviæ*, 1837. 4to. pp. 38.

*Contributions on the subject of the Sanguineous Tumour of the Skull of New-born Infants.* By J. A. BURCHARD, M.D., Lecturer on Medicine in the University of Breslau, &c.—*Breslau*, 1837. 4to. pp. 38.

WE hail the appearance of M. Valleix's book with real pleasure. For, if it be true—and this will hardly be questioned—that the existing knowledge of infant pathology is lamentably deficient, it is equally certain that we can only hope for its advancement through a system of minute and careful observation, such as that adopted by the school of which the author is a disciple. And though we should have felt much better pleased had his experience furnished him with materials for a complete treatise on that singularly obscure branch of the science, yet, on first view, far from being disposed to object to his researches on the score of their limitation, we fancied we should find in this a source of satisfaction. We looked upon it as a sort of mute guarantee of the soundness and good faith with which he had elaborated the subjects undertaken. We presumed that M. Valleix had wisely preferred the really useful task of establishing truth on a few points to the empty—though unfortunately too much prized—honour of copying from others, or of speculating through the entire range of a subject. Whether our first impression has been strengthened by intimate acquaintance with the book will appear as we proceed.

The author gives considerable evidence of what is always cheering to meet with—earnestness of purpose. In the opening chapter of the volume will be found a careful description of his manner of observing. This is an almost completely novel, and certainly a most gratifying feature in pathological works. It shows that scientific men are not now contented with the mere fact that those works are ostensibly founded on bed-side observation, or that a number of cases are scrupulously detailed in them: they require, in addition, an account of the method and instruments by which the alleged facts were obtained. It is not very long since the act of observation was looked on as work fitted only for drudges. Systematists who, like Pinel, condescended to allow that established facts were really necessary materials in the construction of doctrines, were still contented to allow their cases to be collected by their pupils. But the qualifications for observing are now admitted to be of a different order. The labours of Louis, more especially, have shown what observation really is, and what may be effected by its aid; and have consequently raised it to its just eminence as an intellectual pursuit. And it will be admitted that if minds of severe character are justified in demanding an account of the means used in observing, and of the precautions taken for the avoidance of error, when the subjects examined are adults, such an account is still more necessary in the instance of new-born infants. By the side of these helpless little beings, whose means of expression are so few and so imperfect, the ordinary difficulties of clinical observation are increased tenfold. We have known highly judicious practitioners sink in this, to them, novel situation, into absolute ciphers. We have seen them unable from the mere want of the habit of close and methodic observation, and the impossibility of employing the routine system of question and answer, to which they were accustomed, to draw from the phenomena of the disease any sure notions of its nature. As it seems to us, therefore, M. Valleix has conferred a double favour on the profession by the careful exposition of his mode of observing. He then allows each reader to estimate for himself the degree of credit his statements deserve, and furnishes those disposed to avail themselves of them with the means of obtaining—if we may so speak—the maximum of evidence from the minimum of facts, and hence of easily rising above the degree of accuracy in their judgments respecting infantile disease, to which they could otherwise attain. M. Valleix, however, is not the first who has striven to improve the method of examining infant patients. The excellent work of Billard contains directions full of sagacity and, for the most part, simple in their application, for smoothing the difficulties of that embarrassing task: and the recent volume of Mr. Dendy, noticed in our last number, shows that the profession in this country are no less aware of their magnitude, and eager to lessen it.

As M. Billard remarks, the chief circumstances that render the examination of the new-born infant so puzzling a matter are, 1st, the absence of speech; 2dly, the agitation caused by it; 3dly, the cries that accompany that agitation. But the two latter obstacles are not constantly present; there are periods, though they be rare, in which infants are at rest. These brief moments should be dexterously made use of for the examination of such phenomena as are affected by the agitation of the infant, while the state of the remaining symptoms may be ascertained when the habitual restlessness reappears. Hence a natural division into two classes of symptoms. The first of these should be got through, according to M. V., when the patient sinks into a doze after being fed or washed, &c. At such times the state of colour, the expression and spontaneous movements of the face, the pulse, the heart's pulsations, the number of inspirations are the chief points for investigation. The naturally calm and expressionless character of the face is from the first changed by disease: the features contract, rugæ appear in the forehead, the eyebrows are drawn near each other, and the labial commissures carried outwards. In pneumonia M. V. found this altered state of the features persistent; in aphthæ (muguet) intermittent. In the latter affection he plausibly ascribes the facial contractions to intermittent colic, especially as he has observed them to cease at the same instant as a copious alvine evacuation took place. But the *same* expression appears, according to our author, under every variety of suffering; nor does observation bear out the statements of Jadelot as to a special cast of physiognomy distinguishing each particular disease. We confess we were always inclined to look on the descriptions of Jadelot as somewhat visionary, and are gratified to find our opinion justified by the authority of M. Valleix.

M. Valleix furnishes us with some interesting results regarding the *normal* frequency of the pulse during the first days of existence. Few practical men can have failed to ascertain for themselves the extreme difficulty of accurately counting an infant's pulse, and none can have been contented with most of the expedients advised for facilitating the process, such as putting the infant to the breast, slipping the finger within its mouth, &c. These and similar contrivances are either useless as means of quieting the little patient, or, what is still worse, from their nature quicken the heart's action. A very convincing proof of the difficulty of the attempt appears in the fact that in many of Billard's cases no mention is made of an examination of the pulse, while he states that in others where this was examined its normal frequency varied from *eighty* to *one hundred and eighty* beats in a minute! M. Valleix's method, consists merely in gliding the finger on the radial artery, while the infant dozes, and following without controlling whatever movements it may make. This simple plan succeeded so well, that in thirty-four cases the author was enabled to count the pulse eight or nine times out of every ten attempts. We must, however, regret the very small number of subjects he found in the fit conditions of perfect health and quietude for its application. His observations were made on thirteen infants only, aged from two to twenty-one days. The lowest number of beats was 76, the highest 104, the mean 87. We do not contest the accuracy of these results; although they will be found to differ very materially from those of Mr. Gorham noticed in a former number of this journal, (Brit. and For. Med. Rev., Vol. V. p. 269.) The results in the following table relating to subjects aged from seven months to six years approach very closely to those of Mr. Gorham.

| | Group of oldest Subjects. | Group of youngest Subjects. |
|---|---|---|
| Number of subjects | 3 | 30 |
| of observations made on them | 25 | 316 |
| Mean age | 64.7 months. | 17.17 months. |
| Mean number of pulsations | 108.31 | 124.47 |

M. Valleix rejects, and on fair grounds, the auscultation of the heart for the purpose of ascertaining the *frequency* of pulsation: the act of examination inevitably irritates the infant. In a note to this paragraph is a curious document by M. Lediberder relative to the frequency of the pulse previous to the section of the funis. In six examinations made the first minute after expulsion he found the mean number of double pulsations 83.3. This average augments rapidly, for in sixteen infants auscultated after the third or fourth minute of extra-uterine life it equalled 160. How much of this increased rapidity should be attributed to normal change in the activity of the circulation, and how much to uneasiness of the subject, it is not easy to determine.

The main obstacle to examination during the second stage, when the infant is more or less agitated, arises from the impossibility of distinguishing cries of impatience from those produced by real pain, whereby the observer is prevented from estimating the tenderness of various parts. The healthiest infants in the world will scream under palpitation of the abdomen, if laid, as they usually are, on their backs. We have, it is true, seen a plan employed with some success by M. Ricord for quieting their clamours, that of seizing the infant by the chin and gently rolling the head from side to side; but the calm thus produced lasts for a moment only. M. V. appears to have been more fortunate in his contrivance. Availing himself of the eagerness with which infants stare at a strong light he supports them in a sitting posture opposite a window. Their attention, he says, is almost immediately riveted on the light, nor will pressure call it away if there be no tenderness present. Such, according to M. V., is the success of this plan, "that he was enabled in various cases, in those of aphthæ for example, to ascertain by its help the existence of general abdominal pain or even of local tenderness limited to a small space, such as the epigastrium or right iliac fossa." We sincerely hope that this method may be found as successful in the hands of others, but we confess that we have not found little infants so observant of anything, during the existence of a disagreeable external impression, as to lead us to have much confidence in the plan proposed.

Our author's experience leads him to admit the practical superiority of immediate to mediate auscultation in infantile diseases; and he invariably failed in obtaining satisfactory results with the stethoscope, while the application of the ear was at once decisive.

Among thoracic affections, two only are noticed by our author. The quantity of matter devoted to these is exceedingly unequal; for while 158 pages are occupied in describing pneumonia, the history of pleurisy is despatched in a few lines. The reason of this will appear hereafter. In analyzing the copious details on pneumonia we shall give the greater part of our space and attention to the chapter on symptoms, as it comprises the most practical information. We shall only notice such points of the morbid anatomy as either confirm or form striking exceptions to the laws ascertained respecting the disease in adults. The number of cases on which the article is mainly founded amounts to fifteen only, collected, as all others in the volume, at the Foundling Hospital at Paris. The evidence of one hundred and fourteen others, supplied by M. Vernois, is, however, brought to bear on a number of important questions. It is necessary to state also, that three only of M. Valleix's patients were affected with simple pneumonia; in the others the disease was complicated either with aphthæ, œdema, erysipelas, or tubercles. The subdivisions made in consequence of these complications reduce so materially the numerical value of the results, that they lose all shadow of a claim to be considered as expressing general laws.

A notable difference between the infantile and adult lesion presents itself early in M. V.'s description. The section of the diseased tissue was never *granular* in the former; on the contrary, when the substance of the lung was indurated it presented, on division, a perfectly smooth, clean, and shining surface, resembling polished marble; when softened it lost its clean character somewhat, but retained its other peculiarities. Even when *torn*, as it was in one case, no sign of granulation was produced. Moreover, the morbid parts, instead of being, as in the hepatization of adults, friable and easily penetrable by the finger, occurred in the form of hard and resisting nuclei. At least such was its condition in twelve cases out of fourteen; in the remainder, the diseased parts were either softened or consisted of a mixture of softened and indurated substance. These peculiarities, added to the *total* absence of crepitation, its *rapid* precipitation in water, the deep violet hue of the expressed liquid, seem to prove strong dissimilarity between the hepatization of infants and adults; while, on the other hand, there is an almost perfect resemblance between the anatomical state noted by M. V. and that described by Louis under the name of *carnification.*

The comparative rarity of double pneumonia in the grown subject, especially if the lungs be free from organic disease, is well known. The following table shows a new difference between the laws of that affection in early infancy and in adult age. By adding MM. Valleix and Vernois' observations together, we find the number of examples of

| | |
|---|---|
| Pneumonia of the right lung . . . . | 17 |
| left . . . . . . . | 0 |
| double . . . . . | 111 = 128. |

Of these 111 double cases, the disease predominated

| | |
|---|---|
| on the right side . . . . . . . | 59 times, |
| on the left . . . . . . . . . . | 10; |
| and was equal on both . . . . | 42 = 111; |

while, as regards the part of the lung affected, (doubling M. Valleix's numbers so as to count each lung as a distinct case,) there appear of

| | |
|---|---|
| Pneumonia of base and summit . . | 44 examples, |
| base alone . . . . | 44 |
| summit alone . . . | 20 |
| Disseminated lobular pneumonia . . | 31 = 139. |

A similar predominance on the right side exists in the adult, though not to so great an extent; but in the adult the summit is much less frequently affected than in the ratio deducible from the last table. Indeed, as M. Louis has shown, primitive pneumonia of the upper lobe is, in the adult, almost wholly confined to subjects aged upwards of forty-five; a fact which fully accounts for the greater mortality from the disease when seated in the upper lobe.

The other lesions found in the parenchyma of the lungs were vesicular emphysema in three cases, and tubercles in one. In the tuberculous subject only were there any traces of pleural inflammation apparent; and in this case, judging from their locality and age, they were unconnected with the pneumonia. In 123 cases which fell under the notice of M. V., pleurisy occurred only twenty times; a fact which proves, contrary to the opinion of many, the extreme comparative rarity of pleuropneumonia in infants.

The Symptomatology next demands our notice: and here the subdivision of cases renders anything like close analysis impossible. We can only allude at all fully to the symptoms of the *simple* disease, glancing incidentally at any very wide departure from their ordinary train in the complicated cases.

Foremost in the list come difficulty of breathing and hurried respiration; the former having existed in ten, the latter in eight, out of fourteen cases: the intensity of these symptoms was proportional to the extent of disease; the inspirations were short, deep, and precipitate, and attended with elevation of the shoulders, averaging forty-four or fifty-eight in a minute, or so rapid as to baffle the attempts made at counting them. On the other hand, in the cases complicated with œdema, the frequency of respiration was below the natural standard. Its most striking character, however, and one which exists in simple œdema neonatorum, was extreme irregularity.

According to our author, the cough ranks high as a diagnostic sign; and this because of its frequency of occurrence, and its "belonging especially to pneumonia." That it was frequent, appears from its having occurred in ten out of fourteen, or, more correctly perhaps, out of twelve cases; but where is the proof of its being peculiar to pneumonia? We find none in the pages before us: they contain no attempt at distinguishing it from the cough of bronchitis, or tubercles. Our author possibly means that this symptom was peculiar to pneumonia, as compared with œdema and aphthæ; a proposition few will be inclined to dispute. Be that as it will, the cough (in the simple cases) occurred in short paroxysms, ceased the last day of life in two instances, and was not noted the five last in the remaining one. That the hepatization continued to spread notwithstanding the cessation of this symptom, was proved by the physical signs; a fact, by the way, of high practical interest.

M. Valleix remarked in several cases the issue of a slightly sanguinolent, thick, and viscid froth from the mouth; and, in eighteen out of twenty-two pneumonic subjects, M. Vernois, to whom he communicated his observation, noticed a similar phenomenon. This froth is evidently a bronchial product, as M. Valleix found a considerable quantity of similar matter in the trachea and bronchi of all subjects attentively examined after death.

The importance of percussion, as a sign of the growth and progress of inflammation of the infant lung, is scarcely inferior, in M. Valleix's judgment, to that which it possesses in the adult. It appears that, in every case of simple pneumonia, complete dulness, or at least obscurity, of sound, contrasting strongly with the sonorousness of the healthy parts, existed in some quarter of the chest. It is true, as we are told, the extent of this dulness was uniformly more limited than that of the hepatization, as disclosed by the inspection after death; but this disparity is not to be wondered at; for the rapidity with which the disease spreads is so great that a few hours suffice to change notably its boundaries. Thus, a remarkable case is given at length by the author, in which the chest is noted as being "of normal and everywhere equal sonorousness, the respiratory murmur pure and vesicular," on the 10th; whereas, on the 11th, "the whole posterior aspect of the right side gives a dull sound on percussion, as well as the inferior third of the left. A distinct bronchial souffle is heard over the same extent of surface." (p. 124.) Here this extensive hepatization (and it still further increased in the seventeen hours intervening between the last examination and death,) required only twenty hours at the most for its production. Such great strides may well make the practitioner tremble.

But, on the other hand,—independently of the lobular form of pneumonia, wherein the absolute inutility of percussion is granted by M. V.,—when the lesion is superficial, the employment of that instrument of enquiry is ineffectual. In one case of this kind, his favorite method is exculpated on the score of the disease having been completely latent; but we cannot forget that its prime value in the adult consists in its making manifest the presence of the malady when all rational symptoms

(as, we admit, very rarely occurs,) are wholly wanting. M. Vernois found considerable dulness in twenty out of twenty-two cases; slight obscurity in the remaining two.

Our author has employed percussion in further investigation of the question concerning double pneumonia, to which we have already alluded. His cases go to show that the disease is very rarely double *primitively;* that the seizure of the two organs is successive, not simultaneous. He adds, "In truth it is only on the approach of death, and when it has gone through its periods for a long time in one lung, that pneumonia attacks the other; and the inflammation of the latter, which is almost always very limited in extent, may even be regarded as a secondary lesion." (p. 134.) There is considerable vagueness about this statement; nor is it easy to discover whether its author meant it to apply to adult and infant pneumonia both, or to the latter alone. Admitting either supposition, the phrase "for a long time" stands unsupported by evidence, and is, we strongly suspect, incorrect. How can long periods, in the infantile disease at least, be spoken of, when its rapidity of progress is such as results from our author's own researches?

Among the auscultatory phenomena, the subcrepitant and crepitant rhonchi are enumerated, but allowed no great importance, as diagnostic signs. The former was totally wanting in some very severe cases, and usually appeared, when it did exist, during the last days only. The crepitant rhonchus was heard but twice: still it is not to be wholly disregarded as a sign. Indeed, its rarity alone seems to deprive it of first-rate value in this respect; for in both these cases it was the sole, or almost the sole phenomenon indicating the existence of pneumonia. To us the subcrepitant rhonchus seems too lightly passed over; for our author's analysis establishes its presence in ten cases, at least, out of fourteen. Situated in all of these at the postero-inferior two thirds of both sides of the chest, even where the pneumonia was limited to one lung, it possessed the special characters of that subcrepitant rhonchus pathognomonic of acute capillary bronchitis in the adult.* Hence it seems probable the parenchymatous inflammation took place in some instances by extension from the smaller tubes. Of this, however, we are not by any means certain; inasmuch as the infant tissue differs in structure sufficiently, for aught that is known to the contrary, from that of the adult, to cause the production of a different form of rhonchus under similar morbid conditions of the vesicles. M. V. leaves this question untouched, and is rather obscure on the matter generally; for in this section he speaks of the subcrepitant rhonchus as a sign of pneumonia, while afterwards (p. 172) he evidently wishes us to understand that he had ascribed it to bronchitis, and allowed that in the infant, as in the adult, it distinguishes that affection from inflammation of the pulmonary tissue. For our parts, admitting, in these cases, the conversion of one disease into the other by simple propagation of the morbid action along the continuous surface, we see nothing therein clashing with Louis's announcement that pulmonary catarrh is not observed to pass into pneumonia, for the simple reason that his statement referred to the adult alone. A tracheal rhonchus existed in four cases, in two of which it supervened along with and

* Vide British and Foreign Med. Review, No. XI., p. 36.

at the same time as the local symptoms. As it existed only in the severest cases, it may be classed among the signs most to be dreaded. This observer did not trace any very distinct connexion between it and the bronchial spumous matter already described. In every instance where the dull sound was tolerably extensive, the respiratory murmur was either diminished in force or replaced by a bronchial souffle; and well-marked bronchophony coexisted with both these conditions.

The febrile phenomena are next discussed, and among them the state of the pulse deserves mention. In the simple cases it grew very rapid on the appearance of the first local symptoms; no acceleration took place in the œdematous infants; while in those affected with aphthæ it rose greatly on the eve of the earliest symptoms developing themselves, and then fell. When rapid, it was also strong, full, and hard: but, to whatever degree they had previously existed, it invariably lost all the characters of reaction some time before death. This last peculiarity is, according to M. V., common to all the affections of new-born infants. In the simple cases, the heat and anxiety keep pace with the increased frequency of the heart's action; in the others they underwent no change. From these facts, and a variety of minute discussions on some other febrile symptoms, M. V. concludes that "the importance of carefully distinguishing simple from complicated pneumonia is clearly shown: for high fever exists in the former; whereas, if slight quickening of the pulse appear in the latter, as it is far from always doing, it lasts only for some hours previous to the development of local symptoms: and it is equally clear that the period of the affection at which the patient is examined must, in reporting cases, be accurately recorded. For, if the examination be delayed to an advanced period, no fever, but a general collapse, will be observed; whence the easy conclusion that febrile reaction is wanting in the parenchymatous inflammation of new-born infants." The fugitive character of the fever had its share, no doubt, in betraying Billard into the latter erroneous conclusion.

We pass over the symptoms connected with the digestive organs, and arrive at the sections referring to the duration and prognosis of the disease. In the simple and œdematous cases, the mean duration was three days and a quarter; in those complicated with aphthæ, twelve and a quarter. Every one of M. Valleix's patients perished; but, as he informs us with considerable naïveté, "we must not conclude thence that pneumonia is necessarily mortal in new-born infants." We trust not: his cases were almost all complicated with serious affections, and were all observed at an hospital where, as it appears from a crowd of passages in his book, almost every disease terminates fatally. This frightful mortality he considers to depend, in a great measure, on the utter neglect of all hygienic care under which its victims suffer. Can the recovery of these infants in any fair proportion be hoped for, even under the most excellent treatment, when their vital powers have been previously reduced to languishment by the want of such natural excitants as exercise, cleanliness, and breast-milk? That the disease is highly dangerous we question not for a moment; and, until a series of observations be made under juster conditions than the present, this we believe to be the safest and most correct conclusion.

The mean age of these patients at the moment of seizure was about ten days; two days and twenty days being the extremes. M. Valleix has not met with any cases corroborating Billard's notion that pneumonia may be developed during intra-uterine life. Both sexes appear equally liable to the disease; its greater frequency in the male in after-life,* no doubt, originating in his mode of life. General debility appears to act as one of its exciting causes: upon the influence of temperature, and its changes, no information of a decisive kind is given. Such are the etiological inferences of our author, and meager enough they are.

The frequency of the disease at the Enfants Trouvés is always very great; but it may occasionally appear even as an epidemic. At least, of 114 infants opened by M. Vernois, without selection, during the months of February, March, and April, 1837, 113 presented hepatization of one or both lungs.

M. Valleix is right in saying that, "in cases where we possess no positive knowledge, probabilities are valuable; that it is better to take these as guides in practice than have recourse to mere random and chance essays;" and for this reason we by no means contemn his struggle to obtain a ray of therapeutic light from the analysis of the only five cases in which "any treatment of the pneumonia was undertaken." The light thence derived is, however, far from being dazzling; and it will quite suffice for us to say that the employment of leeches and tartar emetic, of opiates and emollient drinks, are recommended. Blisters, in his opinion, and in ours too, should be erased from the list of remedies for the diseases of new-born infants. Those who desire further detail will find an abundance in the present section, but, as we believe, nothing really novel.

Pleurisy is shown by our author's experience to be a rare complaint in the new-born infant. It occurred in one eighth only of the pneumonic cases collected by him and M. Vernois, and in no single instance did it exist alone. In consequence of this, but especially on account of his possessing no accurately detailed examples of the affection, he does no more than allude to its existence.

The next 260 pages are devoted to the history of a disease but little known in this country, the *muguet* of the French, the *milk-thrush* of our own writers. Many practitioners, indeed, are not aware of the distinction between it and common aphthous ulceration; yet, so early as the time of Amatus Lusitanus, their difference had been acknowledged, while to the French observers of the present day we owe the clear establishment of their total dissimilarity. This they have shown, not only as regards the local appearances in the mouth, but also their various other phenomena: aphthæ being a greyish ulceration, never attended with formation of true false membrane; while *muguet*, or milk-thrush, consists of pseudo-membranous matter exuded from the mucous surface, without the smallest trace of abrasion. Among these observers, Guersent, Lélut, and Billard deserve particular mention. The German pathologists, too, have evidently recognized the disease; and, so far as the description of the adventitious product goes, one of their number, Jörg, gives tolerably accurate information.† Doepp, physician to the

* At Paris, the ratio of pneumonic males to females is about as 3 : 1.

† Handbuch der Kinderkrankheiten, s. 498.

St. Petersburgh Foundling Hospital, states, in a statistical report, that scarcely a single infant admitted into that institution escapes *aphtha:* he adds, that it appears in the form described by Guersent and Billard under the name of *muguet;* that he has always seen it as a symptom of other diseases; and signalizes the error of those writers who ascribe to it a *vesicular* form.* The gravity of the two affections differs so enormously at Paris and St. Petersburgh, that it seems fair to doubt their identity: at the former place *muguet* cuts off five sixths of those attacked, while at the latter it is "a very harmless complaint." The Russian practitioner is not of this opinion, however, and, with amusing self-complacency, attributes the innocent character of the disease among his patients to the treatment adopted; and mainly to his spurting breast-milk frequently into the mouths, while the French deprive the infant of that fluid. (*Op. cit.* s. 153.)

The distinction is formally admitted, too, in the recent work of Drs. Evanson and Maunsell. Though the chapter devoted to it is a mere abridgment from Billard, yet they have evidently themselves seen the disease, and even observed it so far advanced that "the white fluid exuded from the nostrils;" a state which M. Valleix does not appear to have witnessed. But, in perusing most of the writings on this affection, it becomes manifest that their authors, though many of them admit the frequency of intestinal disorder as a complication, view it as a *local* disease only. It plainly appears that their attention has been engrossed completely by the buccal lesions. Now, according to M. Valleix, "it is not simply an affection of the mouth, but a disease having a much more extensive seat in the digestive tube, and giving rise to numerous secondary lesions." (p. 203.) This important conclusion, to which we shall gradually lead the reader, is drawn from the analysis of twenty-four cases of unobjectionable exactness.

The following is an abridged account of the general course of the disease. The age of the patients was less than one mouth, and the malady did not in any instance continue till the end of the second; their mean height was one foot six lines and a half (Fr.), their strength good. With one exception, the complaint was developed *in the infirmary*,† without any evident contagious influence. All the cases occurred in the months of July, August, and September. In seventeen out of twenty-three subjects, erythema of the buttocks and thighs were the first symptoms, preceded the appearance of the false membrane in the mouth by a mean term of six days and a half, and was followed by yellow diarrhœa; which latter appeared in eighteen cases before the buccal pellicle. The pulse, at first eighty or ninety in a minute, rapidly acquired fulness, and rose to from 116 to 140.

Swelling of the papillæ at the point of the tongue was the first local symptom, and was followed by bright redness of that organ and the rest of the mouth, with ulcerations of the anterior palate. Two days and a half, on an average, after this, the first specks of muguet appeared on the tongue, whence it spread to the cheeks and palate. Slight tympanitis appeared in twenty out of twenty-one cases, attended with abdo-

* Analekten über Kinderkrankheiten, Heft iii. p. 152.

† The importance of this statement will appear by and by.

minal tenderness on pressure, either general, iliac, or epigastric, which was always found to correspond to an intestinal lesion of more or less gravity; vomiting took place in five subjects; in twenty, the malleoli or heels ulcerated. The close was marked by collapse; the pseudo-membrane diminished in quantity; a sort of sub-inflammation took place in various superficial parts, or abscesses formed in their sub-cutaneous tissue.

The mean duration of the disease in the fatal cases was seventeen days and a half; in the two instances of recovery, sixteen and a half.

No trace of organization was ever discovered by M. Valleix in the membranous product; nor was it in any case united to the subjacent mucous surface by the smallest vascular filament. The change that takes place in its degree of adhesion to the mucous surface (a point noticed by all observers) seems to him an argument in favour of its existing under the epithelium in the mouth. At least, it is easy to conceive such position would render it at first closely adherent, while its subsequent looseness would naturally follow the rupture of the cuticle. (p. 354.) Further on he states that, on close examination, the muguet appeared formed by the epithelium itself, thickened and slightly softened, (p. 355;) but on this we have to remark, that muguet, as he elsewhere informs us, was found, with all its characters, in the stomach and intestines, where no epithelium exists: the latter statement, therefore, can scarcely contain a correct theory of its nature. Again, it was found closely adherent in those intestinal cases: the presence of epidermoid structure cannot, therefore, be reasonably adduced in explanation of its adhesion in the mouth. Moreover, in the œsophagus, the pseudo-membrane, to increase the perplexity of the matter, always appeared "situate *upon* the epithelium." (p. 247.) Is it likely that its structure and relations would differ in the œsophagus and mouth? The truth is, M. Valleix seems somewhat at a loss to determine the nature of the membranous matter; but, for our parts, we incline much more to consider it an exudation upon than a morbid state of the substance of the epithelium. This has nothing to do, be it remembered, with the question of its distinction from aphthæ; the author's testimony on this point is unequivocal: the epithelium was in every instance, and in every part, perfectly free of erosion. This fact is not, as would at first sight appear, irreconcileable with his inclination to regard the muguet as formed of layers of hypertrophous and softened epidermis; for it is fair to suppose that the lowest layer might retain its natural character; but it surely argues strongly against the reality of such structure.

We cannot afford space for any notice of the prolix details regarding the morbid anatomy and symptoms of the affection, and pass at once to a question of real interest, its nature. In order to establish his main proposition, that which predicates the merely symptomatic character of the buccal lesions in muguet, it was necessary for M. Valleix to prove two points: first, that in every instance, without exception, some other organ or tissue was affected; and, secondly, that the symptoms of such affection as invariably preceded the formation of the plastic matter. We have, *inter labores et tædia*, satisfied ourselves that both these positions are demonstrated by the author: the mucous lining of the digestive tube *did* present notable lesions, clearly inflammatory, in every subject; and that diarrhœa and fever appeared before the membranous product, has already been announced in the summary with which this division of our article commences. Such is evidently not the state of things in simple local inflammation of the mouth. But there is another fact upholding M. V.'s doctrine that must not be forgotten; it is, that one well-marked case of muguet occurred wherein no lesion of the mouth was detected, the pseudo-membrane having been deposited in the œsophagus alone. From all this it results that "muguet is a disease characterized, first, by febrile action and enteritis of variable severity; next, by a pellicular inflammation of the mouth: and, lastly, by a similar condition of the œsophagus." The author has, so far, observed no example of the pseudo-membranous inflammation of the mouth as an idiopathic affection, or as a complication of any other disease than those of the alimentary canal. But this is not all: advancing a step farther, he enquires whether the existence of the pseudo-membranous product, either in the mouth, œsophagus, or any other part of the digestive tube, be *necessary*, in order that the disease should be characterized. From two cases fully reported at this stage of the work, he evidently infers the negative. In the first of these every important symptom of muguet existed, with the exception of the false membrane; in the second, a case forming a natural transition from that just mentioned to the ordinary examples of the affection, the pellicular product was formed only during the last two days of existence, at a period when, to all appearance, the malady had run its course: but this decision (and it is, we conceive, a just one,) leads to a new enquiry, namely, wherein lies the difference between muguet and enteritis? Three cases of simple enteritis, faithfully recorded in the author's pages, answer this enquiry. They show that in that disease the same lesions, and the same important symptoms, regularly occurred as in the affection known, from its outward visible sign, by the name of muguet; and which sign, therefore, constitutes a mere symptom, and of second-rate importance, instead of the essence of the affection.

The fundamental identity of the two affections being admitted, it becomes a natural question to determine why the membranous formation should take place in some cases of enteritis rather than in others. On this point there is little to satisfy curiosity in M. Valleix's book. He assimilates the buccal exudation in muguet, as others have done before him, to the pseudo-membranous matter formed in the mouth of adults in the course of some acute and chronic diseases; adding, that "its great frequency in new-born infants is accounted for by the weakness of the subjects; for accurate research has proved that debility strongly predisposes to that species of lesion." (p. 487.) We can, however, discover no evidence of the pure enteritic subjects having been gifted with stronger powers of resistance than the sufferers affected with the pseudo-membranous exudation. To us it seems clear that the crowded and ill-ventilated state of the wards at the Enfants Trouvés, and the great neglect under which their inmates labour, act the chief part in giving this character to enteritis. Of the twenty-seven cases of the disease observed by M. V. in that asylum, twenty-four were, as we have seen, attended with pseudo-membranous inflammation; whereas, in private Parisian practice, muguet is almost unknown, and in this country it is equally uncommon. There is no proof of its transmission by contagion. The use of the feculæ as food is reckoned by our author one of the most influential causes of the disease; an opinion that tallies to a certain extent with that of Jörg. That writer, indeed, informs us that, so frequent is the disease, from the very general use of improper food during the four or five days subsequent to birth, and antecedent to application to the breast, that it is only of late the Germans have been persuaded it is not a necessary attendant of that period of life, and been taught that the infant may escape it through proper dietetic treatment.*

The affection is not known to attack infants of more than two months old; and, as may be collected from the following table by Billard, the hottest months of the year are those in which it chiefly flourishes.

| | | | | | |
|---|---|---|---|---|---|
| January, February, March, of | 290 | patients, | 34 | were affected with muguet. |
| April, May, June . . . . | 235 | — | 35 | — |
| July, August, September . . | 213 | — | 101 | — |
| October, November, December, | 139 | — | 48 | — |

Thus, it formed one half of the amount of disease during the three summer months, and about one sixth only during the others; besides, the number of cases in the former period (101) nearly equal that (117) in the remaining *nine* months of the year. The amazing frequency of the disease, (for, adding the above numbers to that of the infants observed in M. Baron's wards in 1834, we have 1574 patients, of whom 358, or somewhat less than a fourth, were sufferers from it,) coupled with its summer predominance, and contrasted with its rarity in private practice, shows that its existence must be attributed almost wholly to the internal arrangements of the hospital in which it occurs. Its fatal character is frightfully established: of 193 cases, 153 terminated by death!

The author's general conclusion on the treatment is, that "the most energetic means are ineffectual, when the infant is not withdrawn from the influence of those causes through which it contracted the disease: in the contrary case, the most simple treatment is crowned with success." (p. 455.)

Proceeding to the Diseases of the Head, M. Valleix first directs our attention to those bloody cranial swellings of infants to which the expressive term Cephalæmatomata† has been applied. In an early Number (British and Foreign Med. Review, Vol. I., p. 182,) we gave a pretty full account of what was then known respecting these tumours. Some novel facts and specious speculations in M. Valleix's work call for further notice of the subject on our parts. We shall take occasion to notice, at the same time, the second work placed at the head of this article, which is the production of a teacher of medicine and a man of experience. Dr. Burchard's essay is very creditable to his talents and learning: it contains several valuable numerical documents on the subject of which it treats, of some of which we shall avail ourselves.‡

Cephalæmatoma, as understood by M. Valleix, is, from its situation, *sub-aponeurotic*, *sub-pericranial*, or *super-meningeal.* Of these varieties the last, seated between the dura mater and bone, is exceedingly rare, and very little of a satisfactory kind is as yet known respecting it; while

* Op. cit. s. 496.

† From κεφαλη, caput, et αἱματωμα, tumor sanguineus.

‡ The author had evidently not heard of the opinions of M. Valleix, though they were originally published so far back as 1835, in the Journal Hebdomadaire.

the simply contusive character of the first, both as regards its mode of production, its external and internal anatomical characters, agreed upon as they are by all observers, relieve us of the necessity of more extended notice. To the remaining species, therefore, alone,—that in which the fluid is effused between the pericranium and bone,—shall we confine our remarks. To it are undoubtedly referrible also the sanguineous collections described by Dr. Burchard as situate between the two laminæ of the bone, or between its internal plate and the pericranium.

It results from M. Valleix's enquiries, that the external characters of cephalæmatoma vary at different periods of its existence, as follows:—When examined on its first appearance, (that is, a few hours, a day, or even more, after birth,) the tumour is small, free from tension, and the bone forming its base may be easily felt by pressing vertically downwards. The integuments are at this period of a deep red hue, and slightly œdematous; the swelling without pulsation; and the characteristic circular prominence as yet unformed. M. Valleix expresses his belief that Naegele's statement with respect to the existence of pulsation in these tumours is not entitled to much confidence; and we confess that we shared in his belief until we had perused Dr. Burchard's essay. But, as a case is there given at length in which very evident pulsations are stated to have been felt by a number of persons, especially when the infant cried, it seems impossible any longer to doubt their occasional occurrence.

As to the period at which cephalæmatoma originates, or is first detected, Dr. Burchard gives us the following particulars respecting the fifty-three cranial swellings observed in his forty-five patients. They were discovered

| | |
|---|---|
| Before parturition, in . . . . . . . | 2 instances, |
| During or shortly after labour, in . . . . . | 24 |
| The second day after ditto, in . . . . . . | 4 |
| The third ditto ditto, in . . . . . | 15 |
| The fourth, fifth, sixth, seventh, tenth, and eleventh; in | 1 |

In two cases the period was not noted.

The tumour soon, according to M. Valleix, acquires its full growth, sometimes in a few hours; in more rare instances continuing to increase in size for a day or two. When fully developed, the swelling is tense, rounded, accurately circumscribed, fluctuating, its investing skin of natural colour, and free from œdema. It is surrounded more or less completely at its base by a narrow, hard prominence, usually called the osseous ring. This formation, according to Michaelis, always exists, and is pathognomonic of the affection. Others have denied the constancy of its presence; and, among them, Zeller* gives a certain number of cases, admitted on hearsay, in which it was altogether wanting. In our author's patients this production was always found, except when the infant died a few hours after birth; and consequently, as we may fairly deduce from a case briefly alluded to in his work, *before sufficient time had elapsed for its formation.* He further states that, by first placing the finger on the bony ring, and then pushing gradually towards the

* De Cephalæmatomate.—Heidelbergæ, 1822.

centre of the tumour, the surface of the skull underneath may be always felt with ease.

There is a strange divergence of opinion among writers as to the frequency of this affection. Naegele, whose extensive experience none will question, met with seventeen cases only in the course of twenty years' practice. Hoere advances as his belief that it occurs once in every hundred births;* Strewe states that, of 250 infants admitted to the Berlin hospital, not one laboured under it;† M. Baron, physician to the Foundling Hospital at Paris, supposes it to exist in one only of every five hundred new-born infants; and Doepp informs us that, at the St. Petersburgh establishment, thirty cases occur annually, which, calculating from the number of yearly admissions, gives one case in 150 births.‡ Among 1937 infants, M. Valleix detected cephalæmatoma four times only. Dr. Burchard, on the other hand, met with forty-five cases, in hospital and private practice, in the space of seven years: and, to judge from the following calculations, it would appear that Breslau is singled out from the rest of the world for frequent development of this affection; for, among 1402 infants born at the Clinical Institution there in seven years, thirteen were affected with cephalæmatoma; whereas, the frequency of the disease at some other Germanic towns is much lower. Thus, there occurred at

| | | | | | |
|---|---|---|---|---|---|
| Dresden, in | 8 | years, among | 1972 | new-born infants, | 6 cases. |
| Würtzburg, in | 13 | .. | 1992 | .... | 2 |
| Marburg, in | 7 | .. | 910 | .... | 4 |
| Berlin, in | 8 | .. | 1314 | .... | 5 |

Several towns, indeed, are enumerated where no single example of cranial blood-swelling was noted, even during a longer space of time. Thus, Moschner and Kurzak state that no instance of cephalæmatoma is recorded to have occurred in 18,292 births at the Prague hospital; though the state of health, &c. of all the infants is very accurately noted down. To us it seems extremely probable that some of Dr. Burchard's cases were nothing more than examples of the *caput succedaneum* of the German, or *sero-sanguineous œdema* of the French writers.

This observer has made some curious enquiries on the moral, hygienic, physiological, and other conditions of the mothers of his patients. Of his results, however, the only seemingly important one is the following. It appears that the infant affected was the

| | | | |
|---|---|---|---|
| First-born | in 29 cases, | Eighth-born | in 1 case. |
| Second | in 8 | Twelfth | in 1 |
| Third | in 1 | Fourteenth | in 1 |

In four instances, the number of children borne was not ascertained. This great predominance of primiparous women among the mothers of cephalæmatomatous children, which had been already noticed by Busch and others, is explained in some measure, in the present instance, by the fact that more than one half of the accouchements at the Clinical Institution of Breslau were of primiparous females. Dr. Burchard does not mention their proportionate number in his private practice, which sup-

* De Tumore cranii, &c.—Berolini, 1825.

† De Cephalæmatomate, &c.—Giessæ, 1828.

‡ Analekten über Kinderkrankheiten, Heft iii. s. 156.

plied him with twenty-one of his cases. Thirty-four of the infants were males, nine females: the sex of two was not noted.

Before considering the etiology of these tumours, we must put the reader in possession of their anatomical characters, as minutely investigated by M. Valleix; and, in order to understand these, certain peculiarities of the infant cranium, so well described by Haller, must be borne in mind. Among other points, this fact was established by him, that, if the pericranium of a new-born infant be removed, and the skull even slightly compressed, drops of blood ooze forth by a multitude of orifices between its radiated fibres, and by their union form a layer of liquid on its surface. M. Valleix's investigations led him to further results, having most important bearings on the disease under consideration.

"On examining the bones of the skull closely, I saw that the internal table was formed *first;* that numerous vessels, ramifying on its surface and forming a rudimentary diploe, *next* deposited a multitude of osseous fibres, and *finally*, that at a later period a layer of compact tissue, constituting the external table, was produced. Thus there are three distinct stages in cranial ossification. At birth the parietal prominences alone, traversed as they are by the majority of the nutritious vessels, have reached the final stage. And it is not a little remarkable that those very parts of the bone, where at a later period both tables unite into one and all trace of diploe is lost, are the only ones that at birth consist of two distinct tables separated by true diploe. For the first fifteen days of life, or in many instances for a much longer time, the bones attain *the second degree only of ossification*, except at the parietal prominences: they have a well formed internal table and a vascular diploe, but *no external table.*" (p. 515.)

Now it follows from this, the author adds, that if pressure, especially circular pressure, be exercised on the cranium when only advanced to the second degree of ossification, the blood must tend to ooze from its surface. This peculiarity of structure and its consequences are, as may be foreseen, brought into play in M. V.'s endeavour to explain the formation of cephalæmatomata.—But to turn to the anatomical characters of the tumour.

M. Valleix invariably found the scalp in the natural state and the aponeurosis unaffected. The investing pericranium was always thickened, but never ossified as admitted by Chelius. Its internal surface was smooth, polished, and serous-like, instead of being as elsewhere, (that is, in the unaltered parts and even close round the edge of the tumour,) roughened by adhering cellular filaments. At the line of attachment of the pericranium to the osseous ring (*bourrelet*) existed a fimbriated prominent border resulting from the rupture of a thin membrane, that lined the pericranium and invested the denuded skull within the limits of the tumour. The aspect of this pseudo-membrane varied; it appeared to consist either of reddish yellow granules of mucous character, or of condensed cellular tissue, or of a thin cartilaginous lamella. In each instance its cranial and pericranial portions, though distinctly continuous, were of different structure. The osseous "bourrelet" surrounded the tumour completely in four of the cases; in the remaining two it was wanting in the vicinity of the sutures. The bony matter of which it consisted was easily removable with the nail from the surface of the skull, which retained its natural curve underneath. It was of triangular form and applied by its base to the cranium, reaching a maximum height of a line and a half.*

* These dimensions prove its vast activity of growth; the cranial bones themselves are only from a sixth to a third of a French line in thickness at birth.

M. Valleix classifies it with the adventitious products termed *osteophyte* by Lobstein. On some points respecting it the two authors, whose observations are under examination, differ considerably. Thus, for example, whenever M. Valleix found the bony ring imperfect, the imperfection always existed in the vicinity of the sutures; Dr. Burchard, on the contrary, states that he almost always found the superior margin, that next the sagittal suture, the most prominent. Again, Busch who has examined seventeen cephalæmatomata doubts the existence of the bony ring altogether, and attributes its supposed presence to "an optical illusion."

In four cases the contained blood was black and fluid; in another, partly coagulated; in the sixth, mixed apparently with a little pus.

It is not our intention to trouble our readers with a catalogue of the numerous hypotheses that have been successively advanced as to the cause of these tumours. The most important among them, that of Michaelis, ascribing their production to disease of the bone, M. Valleix appears to have refuted by his description of the normal condition of the bones of the fœtal cranium. But something may still be urged in defence of Michaelis's theory. It involves as a postulate the preexistence of the tumour to birth. Now, as M. Valleix allows, several facts seem to countenance this view of the case. In the majority of instances, cephalæmatoma has been observed after *easy* labour; in a case attended by M. Fortin the tumour was discovered in the passage by the finger; Osiander, too, asserts that it may be sometimes felt before the rupture of the membranes; and it has existed in infants extracted by the feet. But these arguments are not, according to M. Valleix, unanswerable. For, he urges, the phrase easy labour is one of vague meaning; and though labours, that last from five to six hours and terminate naturally, may deserve that title, yet even in these the head may have suffered very strong pressure either in traversing the os uteri or in sliding along the pelvis. Indeed, this appears to be fully proved by the fact of sero-sanguineous œdema having very frequently followed such labours. In the case of M. Fortin where the tumour was detected, the head had undergone the pressure of the os uteri. Finally, the mere fact of breech presentation constitutes no proof of the head having wholly escaped pressure; numerous examples of the application of the forceps after the birth of the feet and trunk are on record. The importance of these considerations in M. V.'s theory, will presently appear. Meanwhile, a fact observed by Dr. Burchard deserves to be mentioned here, as it seems to have an important bearing on this question. In 1831, that gentleman removed twenty-seven infants from the uteri of their mothers, who were among the victims of the cholera, by the Cæsarean operation. In one of these fœtuses, he found a tumour seated on the right parietal bone. On dividing it, in presence of two brother practitioners, he discovered to his surprise in place of the bony substance two laminæ which were extended into a small pouch, and contained recent, florid, coagulated blood; in other words, a cephalæmatoma. The vessels of the skull, even the minutest among them, were filled with blood.

In the course of his enquiries on the development of the skull, M. Valleix was struck by the almost constant presence of an ecchymosis between the bone and pericranium in the new-born infant. From the position of this ecchymosis, its form, and its absence in the last born of twins, he concluded that *circular pressure, acting at the centre of the passage traversed by the fœtal head,* must be its cause, and hence the neck of the uterus the compressing agent. But further, when the ecchymosis was of considerable extent, our author always found a layer of cellular substance under the pericranium, and in case of an effusion of fluid blood existing on the skull osseous particles were also deposited. Now this peculiar state occurring with almost unchanging constancy, like cephalæmatoma, on the right parietal, is evidently the first stage of that formation. The same cause acting with varying intensity will produce a simple red coloration, infiltration, destruction of tissue, with effusion of fluid blood, or the real sanguineous tumour. But, as may be enquired, how comes it that cephalæmatomata are so rare, while their cause is, according to this doctrine, in every birth constant and inevitable? M. V. has anticipated this objection, and replies that "the most favorable cases for the production of the tumour are those in which a very large portion of the parietal, to the exclusion of the rest of the skull, presents at the orifice of the uterus, and that such cases are of rare occurrence."

For the train of close and plausible reasoning from which these conclusions are drawn we beg to refer the reader to the original volume. The new explanation is undoubtedly simple and ingenious; but further experience must be had of its solidity before it becomes entitled to general adoption. And this, more especially, because a case of breech presentation in the second born of twins is recorded by Dr. Burchard in which the tumour existed, though it is not easy to conceive how any notable pressure could have acted on the head under the circumstances. Besides, if that observer did really in one case, not to speak of the infant removed from the uterus by the Cæsarean operation, diagnosticate cephalæmatoma before the rupture of the membranes, (and there can be little doubt of the fact, inasmuch as the occurrence took place in the presence of several medical practitioners, to " whose great joy" the diagnosis was proved correct on the birth of the child,) the ingenuity of the explanation will not counterbalance the evidence against it. On the other hand, Dr. Burchard has given us at length the anatomical descriptions of the affected parts in nine infants, and from a careful examination of these, we are bound to say that they go to confirm M. Valleix's doctrine respecting the physiological condition of the bone. We are therefore of opinion that the latter has correctly established the predisposing cause of the affection; but must regard its exciting cause as still sub judice.

With respect to the natural termination of these cases, the only one M. Valleix had an opportunity of observing under the proper conditions ended by the gradual extension of ossification from the osseous ring to the centre; the cure was slow, occupying upwards of forty days. The bone remained thickened. Dr. Burchard found a peculiar cicatrix at the end of a year indicating the situation of the ossification of the outer lamella.

The prognosis is by no means serious; M. V.'s patients it is true died, but they died of intestinal affections perfectly unconnected with the cranial tumour. In respect of treatment, the only debateable point refers to the propriety of opening the tumour, or leaving it to the slow process of natural absorption. If it be large and after a few days' trial apparently disinclined to decrease in size, incision becomes, according to M. V., advisable. The opening should be proportional in size to that of the swelling; a free incision will do no harm, for the extreme vitality of the parts renders adhesion a very rapid process, and exposure of the denuded skull little to be feared. The operator must, however, be careful to keep clear of arterial branches; the division of a mere ramuscule caused fatal hemorrhage in a patient operated on by M. V., and he states that Smellie has recorded a similarly unfortunate occurrence. The system of treatment adopted by Gölis, that of producing suppuration by the seton or by caustic potass, cannot be too strongly reprobated. Dr. Burchard gives a tabular view of the treatment adopted in his cases. He comes to the conclusion, that leaving the tumour to the unassisted efforts of nature, a plan which he at first feared to adopt, is followed by as beneficial results as any of the active modes of treatment devised, excepting perhaps incision with the knife.

M. Valleix is silent on the medico-legal questions to which cephalæmatoma might give rise.

The radiated structure of the parietal bones, the appearance of the sub-pericranial ecchymosis, that of the pseudo-membrane and of the osseous "bourrelet" are well shown in some neatly executed coloured figures. Dr. Burchard has also given us three respectable engravings of the same subject.

M. Valleix next proceeds to treat of apoplexy, of which he enumerates two species—meningeal and cerebral hemorrhage. Of these the former, as is known, usually takes place in the great cavity of the arachnoid. In the cases reported by previous observers the apoplectic seizure took place at the moment of birth; M. Valleix, however, gives one of extreme interest wherein the cerebral symptoms did not supervene till the sixth day after that event. An example of an exceedingly rare state, hemorrhagic effusion into the lateral and third ventricles, is next added. Though imperfectly observed, it is of value in so far as it places the reality of such a lesion beyond a doubt.

Of hemorrhage into the substance of the brain, a still rarer occurrence, three cases are reported. The first of these, observed by M. Vernois, is detailed with great minuteness and serves to show the similarity of symptoms to those resulting from the same lesion in the adult. The only signs of the disease in more advanced age, which seem to have been wanting in this instance, were diminished sensibility and the usual altered condition of the organs of sense. It is not improbable, however, that the difficulty of estimating their state was the true cause of the apparent difference. The paralysis existed on the side of the body opposite the hemorrhage; the seat of the effusion was the same as it occupies most frequently in the grown subject—the corpus striatum and optic thalamus; and the anatomical characters were precisely those of an apoplectic clot and cavity of the same age, about seventy-eight days, in the adult. One of the most remarkable features of the case, symptomatically considered, was the rapidity with which the paralysis disappeared. The paralysed limbs had on the tenth day acquired a certain power of resistance, on the twenty-third their restoration was almost complete, and at the end of the second month the only remaining trace of the malady consisted of a slight traction of the right labial commissure. The infant, however, eventually perished of pneumonia.

In one case the hemorrhage appeared in the form of a number of red spots scattered through a softened part of the substance of the brain, and constituted the anatomical form of apoplexy known in the adult as *capillary*.

These cases are of great interest, and seem to show that cerebral hemorrhage is a far more frequent affection among new-born infants than among those who have lived a few years. The treatment of the disease should, judging from the identity of lesion and symptoms, be based on the same principles as guide the practitioner in combating it in the adult patient.

With our author's fifth chapter, devoted to the analysis of thirty-one cases of œdema neonatorum, we shall not long detain the reader, as it contains little real novelty. M. Valleix regards the morbid state of the respiration and weakened circulation as the proximate cause, and constitutional debility and external cold as the predisposing causes of the disease. A striking proof of the accuracy of this opinion is furnished by the numerical results given by the author. The following table shows at once the frequency of the affection at Paris and the influence of season in its production. Of 644 infants admitted into the medical infirmary of the Enfants Trouvés in 1834, 341 were œdematous, and divided as below among the different months.

| *Coldest Months.* | | | | *Warmest Months.* | | | |
|---|---|---|---|---|---|---|---|
| January | 50. | October | 24. | April | 49. | July | 12. |
| February | 37. | November | 33. | May | 22. | August | 0. |
| March | 56. | December | 36. | June | 19. | September | 3. |
| Total = 236. | | | | Total = 105. | | | |

M. V. would have rendered this table much more useful by giving the total number of patients admitted in each month.

The mortality of œdema neonatorum at Paris is not numerically given. This looks ill, especially as it is incidentally admitted to be "frightful." Yet Palletta affirms that forty-two out of forty-three of his patients recovered by leeching.

The work terminates by a short history of two cutaneous affections, "pustules" and pemphigus. A strong conviction, it appears, exists among the non-medical officers of the Enfants Trouvés, that all diseases of the skin in new-born infants are of syphilitic origin. Now, the consequence of this persuasion is that every infant on whose person any one of those maladies appears, and of "pustules" especially the frequency is very great, is deprived of its nurse and transferred to the infirmary. This transference takes place though every function be in its normal state, and the firmness of limb, robustness, and general vigour of the subject be indicative of sound health. Now let the reader attend to the following: "Of thirty-six infants conveyed to the infirmary during the months of July and August 1835, twenty-eight were admitted for "pustules," three for pemphigus. Of these three were after a short stay discharged cured, two were removed to the ophthalmic ward, and all the rest, twenty-six in number, or more than the five-sixths, perished of a disease caught in the infirmary. Twenty-one of them fell victims to muguet." (p. 665.) Now, even admitting that these unfortunates were tainted with syphilis, their deliberate murder must be pronounced unjustifiable; but how shall it be characterized when we learn, as M. V.'s praiseworthy researches prove, that neither of these affections are syphilitic, and that they are of so innocent a nature as absolutely to require no treatment! These facts give an appalling picture of the recklessness with which human life is sacrificed in this institution; and it is with pain we add that we have ourselves at an analogous establishment, the Hôpital des Enfants Malades, observed somewhat similar occurrences. It is a notorious fact that, from one of its wards devoted to the reception of very young infants, scarcely one escapes alive. The little beings recover perhaps from the affection for which they were admitted, but, instead of being then removed at once, they are left to the tender mercies of infection. They vegetate on till they catch some contagious disorder; even from this they may be fortunate enough to recover, but the ordeal is not yet passed, they are left for a third, aye, a fourth, attack to sweep them away. All this we have seen. In the case of the Foundling the mischief originates in the popular prejudice, derived, we believe firmly with M. Valleix, (like all other popular errors in medicine,) from a fallacious doctrine originating with and propagated by professional *theorists.* In the children's hospital, the mortality proceeds more directly from the cold-blooded and calculating atrocity of the parents who procure the admittance of their bastard offspring in the hope of being finally relieved thereby of the burthen of its support. Both cases cry aloud for the interference of the legislature.

With respect to the affections themselves little need be said. The "pustules" described by M. V. differ from the ecthyma infantile of Willan, and indeed from all forms of pustule hitherto written upon. Acute pemphigus he shows to be, when simple, a disease of extreme levity; and makes it plain that the serious cases cited by various writers, as examples of that malady, must really have been instances of some other disorder, in the course of which the bullæ appeared as an intercurrent affection.

Here we close our analysis of M. V.'s work. We have throughout endeavoured to furnish the reader with a just notion of its contents, and to aid in giving the diligence and sagacity of the author the publicity they deserve. It remains for us to notice specially two of its most serious imperfections. Of these the limited number of cases is the most important; the great rarity of some affections such as apoplexy explains, it is true, while it excuses the paucity of adduced examples, but no such plea can be urged in the instances of pneumonia and aphthæ. Secondly, we are not distinctly told what a *new-born* infant is; to what age that term is to be understood to apply. It is only by accident, as it were, and at the 199th page the author acquaints us that to belong to that class the infant must be under two months and a half old. Now there are cases of infants who had passed that age reported in his book, and though the introduction of these may be defended as illustrative of the change that slight advance in age may induce in disease, yet it would, in our minds, have been more natural and more useful to have given a more complete series of cases occurring among the real subjects of his research.

In setting to work on a mere remnant of cases, and in not fixing the age of the subjects to whom the results of his volume may in future be applied, while he occasionally disfigures his pages with loose phraseology, M. Valleix has lost sight of principles which, all will allow, lie at the root of sound and useful research. He is therefore the less excusable for assuming, as he does, a certain tone of conscious superiority, that seems much too frequently to tell the reader "*mihi* summum rerum judicium Dii dedêre, *vobis* obsequii gloria relicta est."

Notwithstanding these drawbacks, which it would be vain to palliate or deny, we have no hesitation in pronouncing the work of such an order, that from its appearance will be dated a new period in infantile pathology.

---

